# New lessons on TDP‐43 from old *N. furzeri* killifish

**DOI:** 10.1111/acel.13517

**Published:** 2021-12-23

**Authors:** Alexandra Louka, Sara Bagnoli, Jakob Rupert, Benjamina Esapa, Gian Gaetano Tartaglia, Alessandro Cellerino, Annalisa Pastore, Eva Terzibasi Tozzini

**Affiliations:** ^1^ Department of Clinical and Basic Neuroscience King's College London UK‐DRI Centre at the Maurice Wohl Institute London UK; ^2^ Bio@SNS Scuola Normale Superiore Pisa Italy; ^3^ Universita' di Roma “La Sapienza” Rome Italy; ^4^ Istituto Italiano di Tecnologia Rome Italy; ^5^ Leibniz Institute on Aging Fritz Lipmann Institute Jena Germany; ^6^ European Synchrotron Radiation Facility Grenoble France; ^7^ Stazione Zoologica Anton Dohrn (SZN) Naples Italy

**Keywords:** amyotrophic lateral sclerosis, animal models, frontotemporal dementia, killifish, neurodegeneration, protein aggregation

## Abstract

Frontotemporal dementia and amyotrophic lateral sclerosis are fatal and incurable neurodegenerative diseases linked to the pathological aggregation of the TDP‐43 protein. This is an essential DNA/RNA**‐**binding protein involved in transcription regulation, pre‐RNA processing, and RNA transport. Having suitable animal models to study the mechanisms of TDP‐43 aggregation is crucial to develop treatments against disease. We have previously demonstrated that the killifish *Nothobranchius furzeri* offers the advantage of being the shortest‐lived vertebrate with a clear aging phenotype. Here, we show that the two *N. furzeri* paralogs of TDP‐43 share high sequence homology with the human protein and recapitulate its cellular and biophysical behavior. During aging, *N. furzeri* TDP‐43 spontaneously forms insoluble intracellular aggregates with amyloid characteristics and colocalizes with stress granules. Our results propose this organism as a valuable new model of TDP‐43‐related pathologies making it a powerful tool for the study of disease mechanism.

## Graphical abstract

Our findings demonstrate that TDP‐43 from the fish *N. furzeri* associates with stress granules, acquires a doughnut‐like nuclear distribution and forms aggregates exclusively in old animals, mimicking what is observed in amyotrophic lateral schlerosis and other TDP‐43‐related diseases.

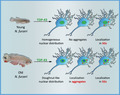

**Abbreviations**
ALS: amyotrophic lateral sclerosis
BSA: Bovine serum albumin
CD: circular dichroism
FTD: frontotemporal dementia
G3BP: GTPase‐activating protein‐binding protein 1
IGTP: Isopropyl β‐d‐1‐thiogalactopyranoside
PBS: phosphate buffer solution
RRM: RNA recognition motif
TDP‐43: TAR DNA‐binding protein 43 kDa
SDS: sodium dodecyl sulphate
SG: stress granule
TEV: Tabacco etch virus
ThT: thioflavin
TIA1: Cytotoxic Granule Associated RNA Binding Protein

## 1 INTRODUCTION

TAR DNA‐binding protein 43 kDa (TDP‐43) is an RNA‐binding protein involved in RNA metabolism (Buratti & Baralle, 2001). It was first described in 1995 as a protein associated with HIV transcription (Ou et al., 1995) and reconsidered later on as an important component of splicing (Buratti & Baralle, 2001) and mRNA transport and translation (Ishiguro et al., 2016; Neelagandan et al., 2018). More recently, TDP‐43 was involved in the relentless motor neuron disease amyotrophic lateral sclerosis (ALS) and in the distinct, but genetically linked, frontotemporal dementia (FTD) (Neumann et al., 2006). Hallmarks of these diseases are the presence of aberrant, polyubiquitinated, and hyperphosphorylated cytosolic aggregates of TDP‐43 in different areas of the central nervous system (Neumann et al., 2006). TDP‐43 aggregates are found in 97% of sporadic ALS and 45% of specific FTD cases. Increasing consensus is that TDP‐43 aggregation is not coincidental but represents the pathological mechanism of these diseases (Hergesheimer et al., 2019).

Stress granule formation is one of the many cellular protective mechanisms as a response to cellular stress (Anderson & Kedersha, 2009). Their formation is initiated by the oligomerization of the core proteins Ras GTPase‐activating protein‐binding protein 1 (G3BP) and cytotoxic granule associated RNA binding protein (TIA1) whose expression is regulated by TDP‐43 (McDonald et al., 2011; Tourrière et al., 2003). TDP‐43 can be found in stress granules (SGs) under cellular stress conditions, and TDP‐43‐positive pathological inclusions in postmortem tissues from ALS patients are positive for stress granule markers (Liu‐Yesucevitz et al., 2010; Parker et al., 2012).

TDP‐43 is a modular protein that binds UG‐rich RNA sequences and composed of a partially unfolded N‐terminal domain linked to two RNA‐binding RRM repeats (RRM1 and RRM2) and followed by an unstructured C‐terminal tail. Until recently, this region was thought to be the hotspot for protein aggregation because it hosts most of the pathologic mutations (Berning & Walker, 2019) and contains a prion‐like sequence (Louka et al., 2020). We have demonstrated that other regions, including the RRM motifs, can play an important role in aggregation and pathology (Zacco et al., 2019). This evidence agrees with a role of RNA in the aggregation properties of this protein (Gotor et al., 2020; Loganathan et al., 2020). We also demonstrated that binding to short RNA aptamers can efficiently prevent TDP‐43 aggregation (Zacco et al., 2019).

Several animal models, from *C. elegans* to mouse, have been developed to permit the study of TDP‐43‐mediated diseases (Liu et al., 2013). One of the limitations of these systems is that either they are invertebrates or, if vertebrates, their relatively slow aging dictates the pace by which studies can progress. Recently, a new vertebrate model organism was introduced that is based on killifishes of the *Nothobranchius* genus, colloquially called annual fishes, and, in particular, the species *N. furzeri* (Cellerino et al., 2016). Killifishes are native to African savannah. Due to the environmental constraints in which this organism evolved to follow the periods of water abundance and depletion, *N. furzeri* matures within 2 weeks (Vrtílek et al., 2018), after which mortality increases rapidly. Also in captivity, lifespan is limited to 3–7 months depending on the genotype (Terzibasi Tozzini et al., 2008; Valdesalici & Cellerino, 2003). The complete genome of *N. furzeri* is available and annotated (Valenzano et al., 2015). Techniques for reverse genetics are available (Harel et al., 2015) making the killifish a system potentially suitable for elucidating the pathologic mechanisms of disease and for screening new compounds. It was indeed demonstrated that the remarkably short lifespan of *N. furzeri*, currently the shortest‐life vertebrate model organism available, recapitulates the main hallmarks of vertebrate aging (Cellerino et al., 2016; Harel et al., 2015). *N. furzeri* is an invaluable tool for studies in disparate branches of investigation such as evolutionary genomics (Cui et al., 2020; Valenzano et al., 2015), regenerative medicine (Wendler et al., 2015), developmental biology (Dolfi et al., 2019; Hu et al., 2020), pharmacology (Valenzano et al., 2006), and ecotoxicology (Philippe et al., 2018). It was also shown that *N. furzeri* presents spontaneous age‐dependent gliosis (Terzibasi Tozzini et al., 2012), neuronal protein aggregation, and loss of stoichiometry of protein complexes in the brain that is triggered by early impairment of proteasome activity (Kelmer‐Sacramento et al., 2020) with selective age‐dependent degeneration of dopaminergic and noradrenergic neurons in the midbrain (Matsui et al., 2019). The fish presents inclusion bodies containing α‐synuclein as those found in Parkinson patients and age‐dependent degeneration of dopaminergic and noradrenergic neurons (Matsui et al., 2019). Genetic depletion of α‐synuclein ameliorated symptoms demonstrating a causal link between aggregation and neurodegeneration.

In the present study, we explored the suitability of *N. furzeri* for the study of TDP‐43‐related pathologies. *N. furzeri* contains two TDP‐43 paralogs, called hereafter Nfu_TDP‐43 and Nfu_TDP‐43L for *N. furzeri* TDP‐43‐ and TDP‐43‐like proteins. We used a complementary approach based on in silico, in vitro, and ex vivo techniques that allowed us to evaluate results both at the cellular and at the protein levels. We compared in silico and in vitro the tendency to bind RNA and aggregate of the *N. furzeri* proteins and their isolated domains with those of human TDP‐43 (Hsa_TDP‐43). We then demonstrated that the *N. furzeri* TDP‐43 paralogs are able to aggregate spontaneously in the intact animal producing inclusions that are similar to those observed in humans. The process is aging‐related as it was detected only in old animals. Finally, we showed that the *N. furzeri* TDP‐43 proteins colocalize in the fish with the stress granule‐contained G3BP, indicating that also *N. furzeri* TDP‐43 is able to segregate in SGs.

Taken together, our data show that *N. furzeri* is a unique model system able to recapitulate most of the in vitro and cellular phenotype observed in TDP‐43 human diseases. This evidence opens the possibility to use *N. furzeri* in ALS studies and provides new insights into the RNA‐to‐TDP‐43 functional relationship.

## 2 RESULTS

### 2.1 *N*. *furzeri* and *H. Sapiens* TDP‐43 have similar phase‐separation propensities

**FIGURE 1:**
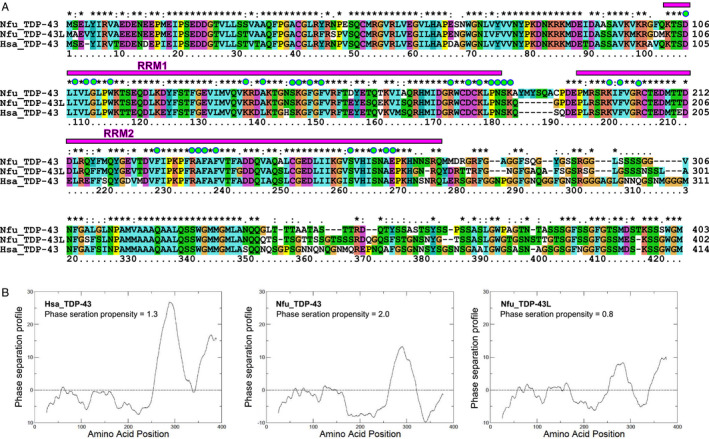
Bioinformatic comparison of *N. furzeri* and human TDP‐43. (a) Multiple alignment of the sequences by clustalx. The positions of RRM1 and 2 are indicated with a magenta box. Amino acids in contact with the RNA aptamer observed in the solution structure (Lukavsky et al., 2013, 4bs2) are indicated with green spots. (b) *cat*GRANULE profiles of the tendence of Hsa_TDP‐43 (left), Nfu_TDP‐43 (middle), and Nfu_TDP‐43L (right) to have phase transitions

Sequence analysis showed an impressive degree of sequence conservation between the *N. furzeri* and human TDP‐43: Nfu_TDP‐43 and Nfu_TDP‐43L share 80% identity with each other, and 75% and 77% identity with Hsa_TDP‐43, respectively (Figure 1a). The homology is however not equally distributed. The N‐terminus and the RRM domains are highly homologous, with hardly any insertion/deletion up to Q269. The only noticeable difference is an insertion of six residues in Nfu_TDP‐43 near the RRM1‐RRM2 interface. More divergent are the C‐termini with a degree of identity of 56% and 59% between the human protein and Nfu_TDP‐43 and Nfu_TDP‐43L, respectively. The C‐termini of the two *N. furzeri* paralogs share 63% identity.

An important property of human TDP‐43 is to phase separate and form SGs. This property is thought to be mediated by the TDP‐43 C‐terminus (Conicella et al., 2016; Louka et al., 2020). Since this is the most divergent region among the three protein sequences, we compared the tendency in silico of the proteins to form granules by the *cat*GRANULE approach (Bolognesi et al., 2016). This software takes into account the structural disorder, nucleic acid binding propensity, and amino acid patterns, such as the presence of arginine‐glycine and phenylalanine‐glycine motifs, to predict the tendency of a protein to coalesce in granules. The program consistently predicted a high tendency to phase separate in all three proteins which, as expected, is mostly localized in the C‐termini with minor differences. The overall *cat*GRANULE scores of Hsa_TDP‐43 and Nfu_TDP‐43L are >1 indicating high‐propensity to form liquid‐like compartments, while Nfu_TDP‐43 has a slightly smaller (0.8) score (Figure 1b).

### 2.2 The C‐terminus of *N. furzeri* TDP‐43 is highly aggregation prone in vitro

We then characterized the aggregation behavior of the isolated domains of the *N. furzeri* proteins in vitro using purified recombinant constructs with the aim of comparing their properties with those of Hsa_TDP‐43. We used the isolated domains rather than the full‐length protein both to be able to appreciate the individual contributions of the different regions of the proteins. We first analyzed the more distantly related C‐terminal domains. The recombinant C‐termini of Nfu_TDP‐43 and Nfu_TDP‐43L (starting at residue Q269) resulted soluble in bacteria and could be cleaved and purified from the SUMO tag although with tiny yields. Conversely, Hsa_TDP‐43 could only be expressed in inclusion bodies as previously reported by independent studies (Shih et al., 2020). Rather than re‐dissolving the protein from inclusion bodies, we decided to excise the glycine‐rich region and produced a shorter fragment spanning residues 315–414 which resulted soluble. This construct did not contain the main hotspot for granule formation but incorporated many of the motifs thought to be crucial in protein self‐association and phase separation (Mompeán et al., 2014; Wang et al., 2018). These constructs could all be produced in suitable concentrations and high purity as demonstrated by SDS‐PAGE (Figure S1).

**FIGURE 2:**
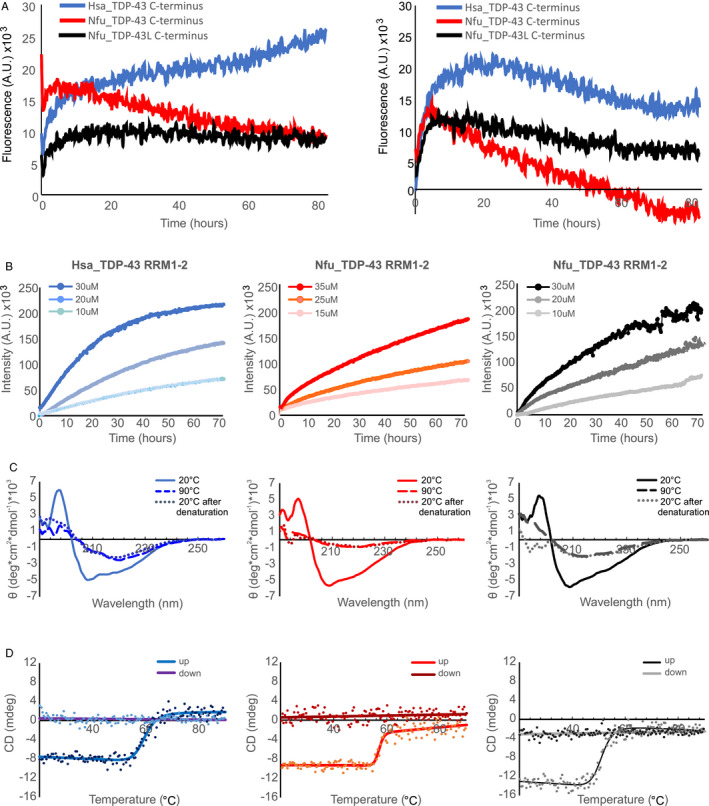
Comparison of the aggregation tendency of the TDP‐43 individual domains. (a) Aggregation ThT assays carried out on constructs of the TDP‐43 C‐termini. Left panel: proteins uncleaved from the SUMO tags; right panel: proteins while cleaving them by TEV protease during the assay (1:10 protein to protease molar ratio). The protein concentrations were 6 µM. Drastic reduction of the signal of Nfu_TDP‐43 indicates precipitation. (b) ThT assays carried out on the RRM1‐2 constructs at increasing protein concentrations. The ThT concentration was 20 µM. (c) CD spectra of the the RRM1‐2 constructs (10 µM) at different temperatures. The spectra were corrected for buffer absorbance. (d) Thermal stability of the three RRM1‐2 domains under the same conditions as in c. The plots are the results of experiments run in triplicates on at least two different batches of the proteins

We measured the kinetics of aggregation following the signal of the fluorescent dye thioflavin T (ThT) detected at 485 nm. Since, however, all constructs resulted anyway little soluble demonstrating a strong intrinsic tendency to aggregation, we carried out the assays in two different ways. We first used the fusion proteins without cleaving the constructs from the tag. Under these conditions, the fluorescent signal of all three proteins increased steeply within the first 2–3 h indicating fiber formation (Figure 2a). After this time, the signal of Nfu_TDP‐43L and Hsa_TDP‐43 C‐termini reached a plateau but not that of Nfu_TDP‐43 whose signal decayed rapidly. This behavior possibly indicated a stronger tendency of this fragment to precipitate. The Hsa_TDP‐43 C‐terminus reached comparatively higher fluorescence values and seemed to have a secondary increase after ca. 60 h. For comparison, we repeated the assay cleaving the proteins from the tag directly in the presence of ThT and followed their fluorescence signals versus time. We observed a qualitatively similar behavior for the three constructs with a decrease of all signals. The signal of Nfu_TDP‐43 C‐terminus decreased appreciably faster.

These data confirm an important role of the C‐terminus in protein aggregation also for the *N. furzeri* proteins in full agreement with predictions. However, the possibility to produce the C‐terminus in a soluble recombinant form using the *N. furzeri* sequences opens new avenues for the study of TDP‐43 aggregation.

### 2.3 The role of RRM1‐2 in the aggregation of *N. furzeri* TDP‐43

Similar experiments were carried out on constructs containing the two isolated tandem RRM domains (RRM1‐2) to assess the contribution to aggregation of regions of the proteins other than the C‐termini and, specifically, of the RNA‐binding domains. Not unexpectedly, RRM1‐2 proved to be more soluble than the C‐termini and could be efficiently produced in appreciable quantities and high purity (Figure S1). Overall, no major differences were observed among the constructs upon aggregation: in all three cases, the fluorescent signal increased as a function of protein concentration (Figure 2b), with aggregation that became progressively visible during the first 20 h of the assay. The curves of the two *N. furzeri* paralogs did not reach a clear plateau within the time of the experiment (3 days). Apart from these minimal differences, the curves exhibited similar shapes with the intensity of the human protein being noticeably higher than that of the *N. furzeri* constructs at comparable concentrations. No difference was observed when the assays were repeated in the presence of salt (15 mM KCl) (data not shown).

These results confirm an involvement in aggregation of the RNA‐binding domains as already observed for the human protein (Zacco et al., 2019) and demonstrate a qualitatively similar behavior for the three proteins as expected from the high sequence homology of this region.

### 2.4 *N*. *furzeri* TDP‐43 RRM1‐2 undergoes an irreversible conformational transition upon thermal destabilization similarly to the human protein

To confirm the presence of a transition toward a β‐rich structure upon protein destabilization which would testify misfolding, circular dichroism (CD) spectra of purified *N. furzeri* RRM1‐2 (10 µM) were measured immediately after the final gel filtration step of purification (Figure 2c). The spectrum at 20°C has two minima at 207 nm and 215 nm, a shape indicative of a mixed αβ conformation. The intensities correlated well with the known structure of this region (Lukavsky et al., 2013) and with previous studies (Zacco et al., 2019). When the temperature was gradually increased up to 90°C, the two minima collapsed into a single shallow minimum at approximately 215 nm as expected for an increase in β‐secondary structure. The spectra remained the same after returning at 20°C indicating irreversibility of the process. We thus assumed that the change of the spectra must be due to a conformational transition that co‐occurs with aggregation.

Thermal denaturation was then recorded following the intensity variations of the band at 222 nm as a function of temperature (Figure 2d). The curves indicated an irreversible transition with an apparent melting temperatures of 57.1 and 50.8°C for Nfu_TDP‐43 and Nfu_TDP‐43L, respectively, to be compared with the melting of 59.6°C for Hsa_TDP‐43. The samples were then extracted from the cuvette and centrifuged at 28000g. An absorbance spectrum between 600 nm and 200 nm of the soluble fraction showed no absorbance at 280 nm indicating almost complete precipitation of the proteins.

The three constructs have thus a similar behavior also in terms of a α‐to‐β irreversible transition of the RRM domains triggered by heat destabilization, although with minor differences in their resilience against aggregation.

### 2.5 Similarities and differences of RNA‐binding properties of *N. furzeri* and human TDP‐43

We then compared the RNA‐binding propensities of the full‐length proteins in silico. We used *cat*RAPID *signature* (Livi et al., 2016), a program based on physicochemical and secondary structure properties and hydrophobicity profiles, to predict RNA‐binding regions. *cat*RAPID *omics* was used to identify RNA sequences able to bind and rank the interactions (Agostini et al., 2013). The program estimates the binding potential of both protein and RNA sequences through van der Waals interactions, hydrogen bonding, and secondary structure propensities allowing for identification of binding partners with 80% accuracy (Lang et al., 2019).

**FIGURE 3:**
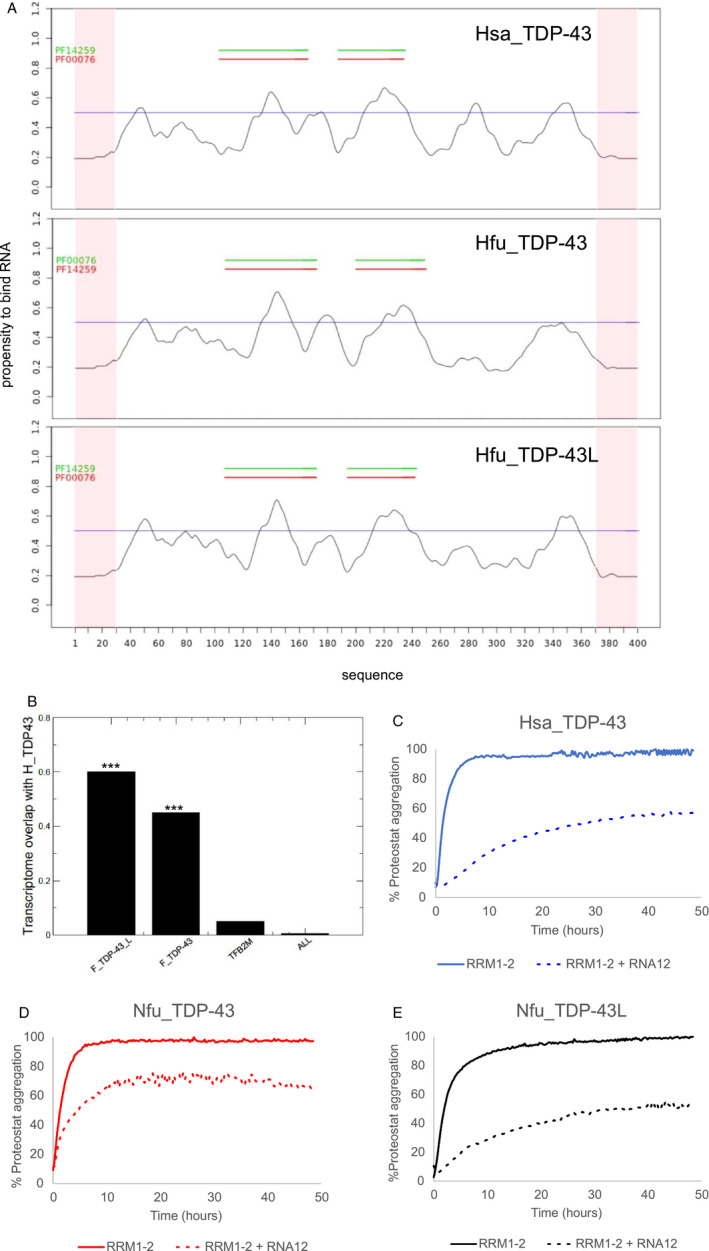
RNA‐binding propensity of *N. furzeri* TDP‐43 sequences as compared to Hsa_TDP‐43. (a) *cat*RAPID *signature* prediction of the RNA‐binding profile along the protein sequences of Hsa_TDP‐43 (top), Nfu_TDP‐43L (middle), and Nfu_TDP‐43 (bottom). (b) The Jaccard index indicates the predicted transcriptome overlap of other proteins with Hsa_TDP‐43. The entire transcriptome ‘ALL’ contains 10^5^ transcripts. The transcripts of Nfu_TDP‐43_L have the best overlap. Nfu_TDP‐43 shows a slightly smaller coverage, while methyltransferase TFB2M, used as a control, has a negligible overlap. (c,d,e) Comparison of the effects of the RNA12 aptamer on the tandem RRM1‐2 domains of Hsa_TDP‐43 (c), Nfu_TDP‐43 (d), and Nfu_TDP‐43L (e) on the aggregation properties of human and *N. furzeri* proteins followed by Proteostat dye assays. The plots in panels B‐F are the results of experiments run in triplicates on at least two different batches of the proteins

*cat*RAPID *signature* predicted similar RNA‐binding profiles along the protein sequences for the three proteins (Figure 3a). Two regions coinciding with the RRM domains consistently exceeded the threshold that defines binding. Overall, however, the profiles of Nfu_TDP‐43L and Hsa_TDP‐43 were more similar, especially over the RNA‐binding domains. Other regions came out just around or above the threshold suggesting the presence of yet unidentified RNA‐binding sites. We then ran *cat*RAPID *omics* against a human transcript library, imposing a high‐confidence threshold (*cat*RAPID scores >2.5). We found 550 transcripts predicted to interact with the human protein. Nfu_TDP‐43L was predicted to interact with 936 human transcripts (out of 10^5^ analyzed) of which 550 were in common with Hsa_TDP‐43. Nfu_TDP‐43 was predicted to interact with the same 550 human RNAs but, in addition, with 719 additional targets. As a control, *N. furzeri* methyltransferase was predicted to interact with just 109 targets of which only 21 sequences being in common with Hsa_TDP‐43.

Overall, the Jaccard index, i.e., the ratio between the intersection and the union of two sets, considering enrichments of shared over total RNA interactions, is 550/(385 + 550) ≈ 0.60 for Nfu_TDP‐43L, 550/(719 + 550) ≈ 0.45 for Nfu_TDP‐43, and 21/(550 + 109) ≈ 0.05 for the control. This tells us that Hsa_TDP‐43 and Nfu_TDP‐43L have more similar propensities than Nfu_TDP‐43 (Figure 3b). A Fisher exact test confirmed that the overlap between the *N. furzeri* and *H. sapiens* TDP‐43 transcriptomes is significant (*p*‐value <0.00001; 10^5^ transcripts used as background). Analysis of the motifs present in the top 100 RNA targets shared between Nfu_TDP‐43L and Hsa_TDP‐43 indicated an enrichment of GU‐rich sequences (CUGG[ACG][UA][CG], UG[CG]UG[ACG][UA], and CUGG[ACG]A) (Agostini et al., 2013) in agreement with the literature (Bhardwaj et al., 2013).

To have some validation of these results experimentally, we checked the effect of an RNA aptamer on the aggregation of the *N. furzeri* RRM1‐2 constructs. We have previously demonstrated that an aptamer (RNA12), known to bind human RRM1‐2 with nanomolar affinities (Lukavsky et al., 2013), is able to inhibit aggregation of this domain almost completely (Zacco et al., 2019). We thus probed the ability of this aptamer on the aggregation of the *N. furzeri* proteins following the fluorescence signal of the Proteostat dye. This fluorophore was chosen for these assays because ThT is known to bind to RNA and to interfere with the measurement (Zacco et al., 2019). Based on the high sequence homology and the specific conservation of the residues involved in interaction, we expected that *N. furzeri* proteins would have RNA‐binding properties identical to the human protein. We found instead that RNA12 has similar inhibitory effects on Hsa_TDP‐43 and Nfu_TDP‐43L but a milder effect on Nfu_TDP‐43, suggesting a lower affinity for the RNA aptamer (Figure 3c‐e), in agreement with our *cat*RAPID *omics* predictions.

These results give us some idea about the exquisite sensitivity by which even small differences in sequence can change the affinity for RNA of these proteins.

### 2.6 TDP‐43 progressively aggregates with aging of *N. furzeri*

We probed the localization of the protein distribution throughout the principal areas of the fish brain, such as telencephalon and optic tectum, performing immunofluorescence experiments on 25 µm cryosections of *N. furzeri* MZM‐04010 brains. We compared animals at 5 weeks, the age at which the animal reaches maturity, with animals at 27 weeks, when age‐dependent mortality starts (Terzibasi Tozzini et al., 2008). We used a polyclonal (Proteintech) rabbit antibody widely utilized in the literature on both human and mouse samples. To make sure that the antibody, that is known to recognize Hsa_TDP‐43, would recognize also the *N. furzeri* proteins, we performed Western Blots both on the purified proteins and on the brain lysate. We found that the polyclonal antibody equally recognizes different regions of all three proteins (Figure S2). Likewise, we were able to identify a band at the expected height between 37 and 50 kDa in a Western Blot performed on the brain lysate (Figure S3).

TDP‐43 staining showed a variable level of diffuse expression in the cell nuclei of young animals, while the presence of doughnut‐like cells (white arrowheads) intercalated between cells with a normal diffuse distribution of the protein (red arrowheads) could be detected only in the old tissue. The progressive plane images taken at a distance of 1 µm (Z1 to 4) underlined the tridimentionality of the perinuclear pathological distribution of the protein around the nuclei (with an average diameter of 5 µm) of the pathological cells (Figure S4). This evidence, together with the presence of cells with diffuse labeling in the old tissue, suggested that the altered protein distribution has a progressive evolution over time.

**FIGURE 4:**
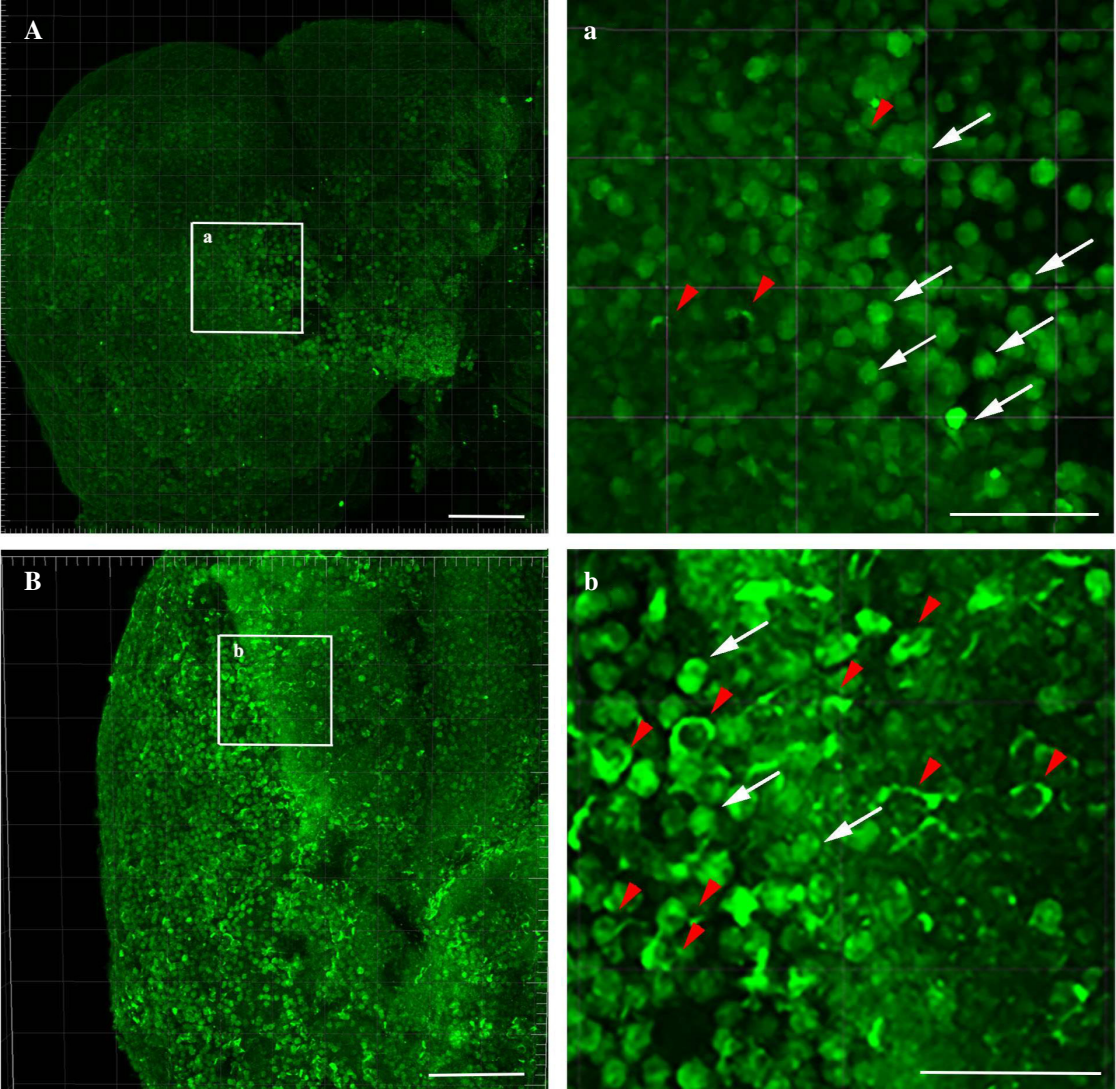
Whole‐mount staining of TDP‐43 in *N. furzeri* brains. Using AbSca/e clarification protocol, we were able to observe TDP‐43 expression in whole‐mount brains of young and old *N. furzeri*. In these images, doughnut‐like cells were clearly visible in old telencephalon (27 weeks, red arrowheads in panel B) as compared to the young animals (5 weeks, panel A). Cells with normal TDP‐43 distribution were present in both young and old tissue (white arrows). Scale bars indicate 50 µm in each panel

To better appreciate the doughnut‐like TDP‐43 distribution in old animals, we performed whole‐mount brain staining (Figure 4 and Videos S1 and S2): we clarified and stained the whole *N. furzeri* brains, by using a Sca/e S immunofluorescence‐optimized methodology (AbSca/e) (Hama et al., 2015), and registered 3D brain reconstructions by acquiring confocal serial stack images and processing them through the IMARIS Software. By comparison of the 3D representations of the tissues from young (Figure 4A,a and Video S1) and old (Figure 4B,b and Video S2) animals, we observed an apparently higher proportion of doughnut‐like cells (red arrowheads) in the telencephalic area of old fishes as compared to that of the young ones. Diffuse‐stained nuclei were detectable in both tissues.

**FIGURE 5:**
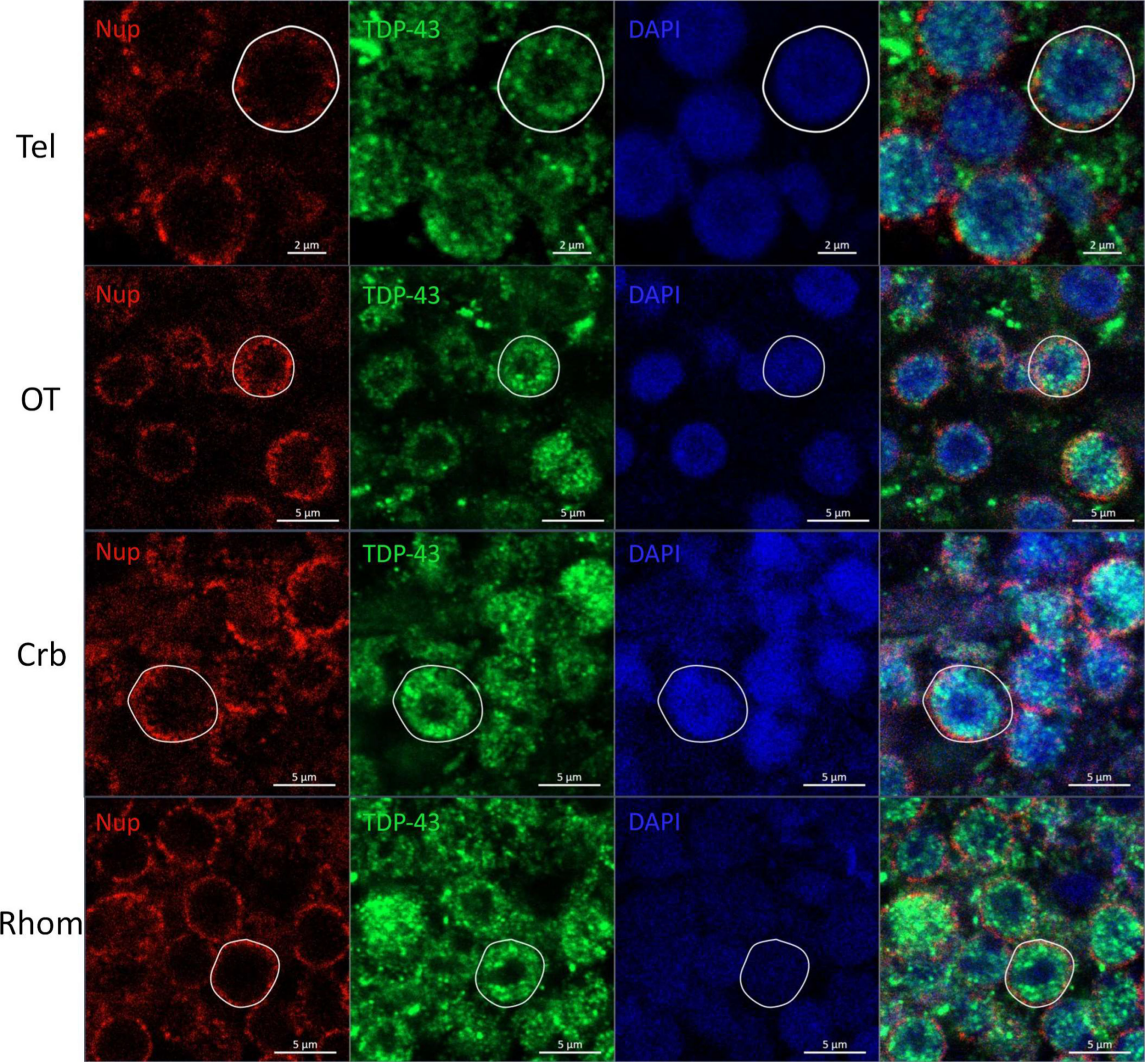
Subcellular localization of TDP‐43 in doughnut‐like cells. Doughnut‐like cells were present in all major brain areas of old animals: telencephalon (tel), optic tectum (OT), cerebellum (Crb), and rhombencephalon (Rhom). The majority of TDP‐43 stain in these cells was located internally to the nuclear envelope as highlighted by the white circle drawn around Nup (nucleoporin) staining. The rest of TDP‐43 staining was cytoplasmatic and partially located in close proximity to the nuclear envelope. The scale bar indicates 5 µm

We then investigated the intracellular localization of TDP‐43 in the doughnut‐like cells via double immunofluorescence using an antibody against the proteins constitutive of the nuclear pore (nucleoporin complex). As evidenced by the white circles around the doughnut‐like cell cores (Figure 5, in both single and double staining images), TDP‐43 distribution (green fluorescence) is mainly confined to the inner side of the nuclear membrane, closely associated to nucleoporin labeling (perinuclear point‐like red fluorescence).

**FIGURE 6:**
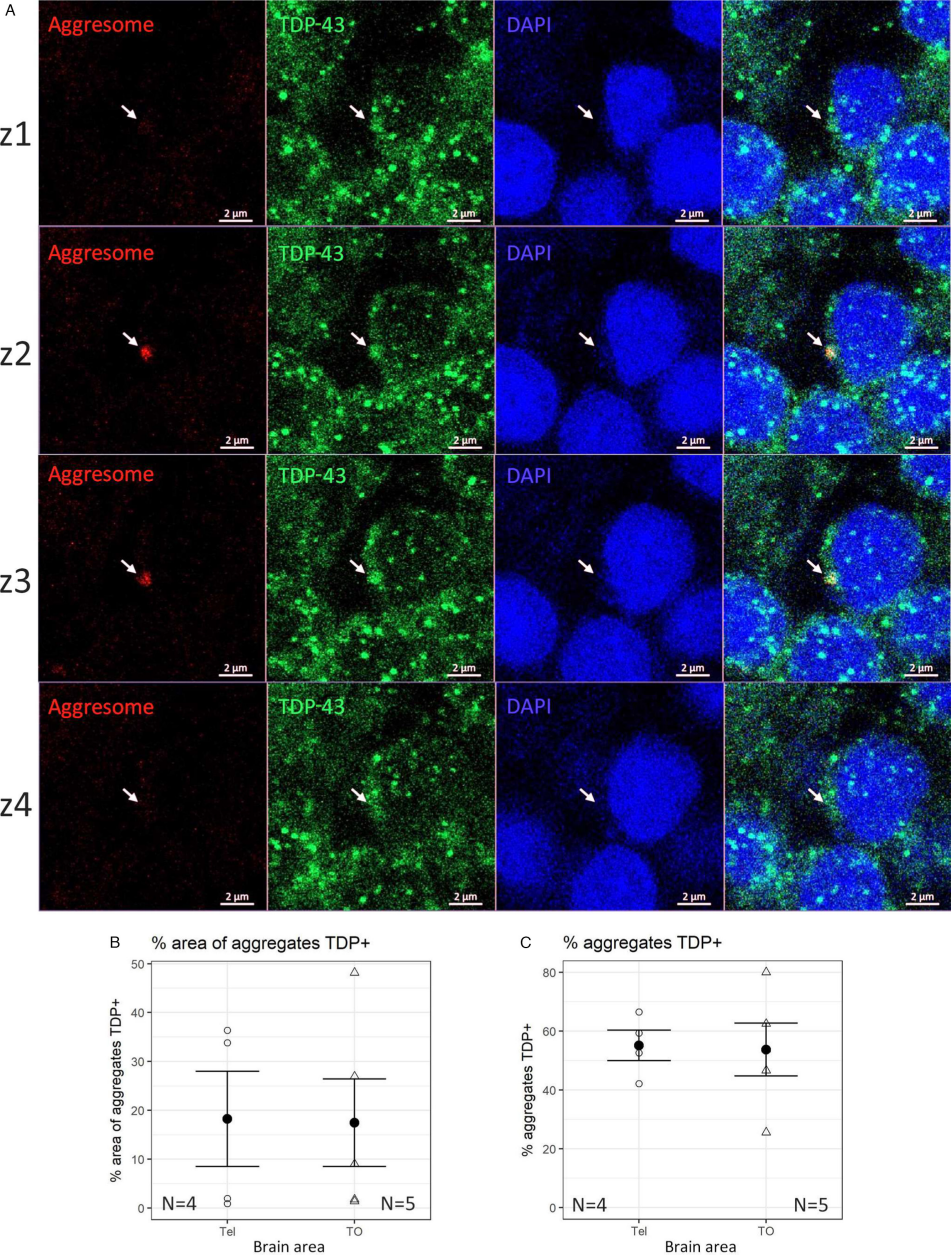
Presence of TDP‐43 in aggregates. (a) No signs of aggregation were found in young animals. In old animals, some aggregates colocalize with TDP‐43 (white arrow). Z1, 2, 3, and 4 represent four consecutive z planes, each acquired at 1 µm step from the previous. (b) Quantification of the area of aggregates labeled also with TDP‐43. (c) Number of aggregates containing TDP‐43 expressed as a percentage. The scale bar indicates 2 µm

We also performed a double staining on brain sections, by combining TDP‐43 immunofluorescence with the Aggresome dye (Figure 6 and Figure S5, green and red, respectively), to discriminate between generic protein aggregates and specific TDP‐43 aggregates, visualized as colocalized green‐red fluorescent dots. Comparison of sections from young and old brain tissues showed the absence of aggregates in young brain tissue, both with and without TDP‐43 staining (Figures S5A,C) and the presence of double‐stained TDP‐43 granules in old animal tissue (Figure 6 and Figures S5B,b,D,d: white arrows). The total lack of aggresome staining in young tissue (Figure S5A,C) indicated that no aggregation is present at this age, consistent with previous results (Kelmer‐Sacramento et al., 2020). We quantified the aggregates in two different areas of the brain, taking into consideration only the old tissues (Figure 6b,c), given that the presence of specific aggregates marker was not detected in young tissues: we chosed the telencephalon and the optic tectum as the brain regions most investigated in teleost experimental models. The presence of aggregates in the killifish telencephalic areas has already been described in previous studies (Kelmer‐Sacramento et al., 2020). Quantification of the colocalization proportion (as expressed both as the area of granules colocalizing with TDP‐43 and the number of granules positive for TDP‐43, Figure 6b,c) did not reveal significant differences between the two regions analyzed, suggesting that the aggregation process could be an age‐dependent cellular mechanism not exclusive to specific types or neuronal areas.

These results demonstrate that it is possible to follow aging‐related aggregation of TDP‐43 in *N. furzeri* mimicking the neuronal alterations typical of ALS/FTD and support the killifish as a convenient model of TDP‐43 aggregation.

### 2.7 TDP‐43 localizes in SGs

**FIGURE 7:**
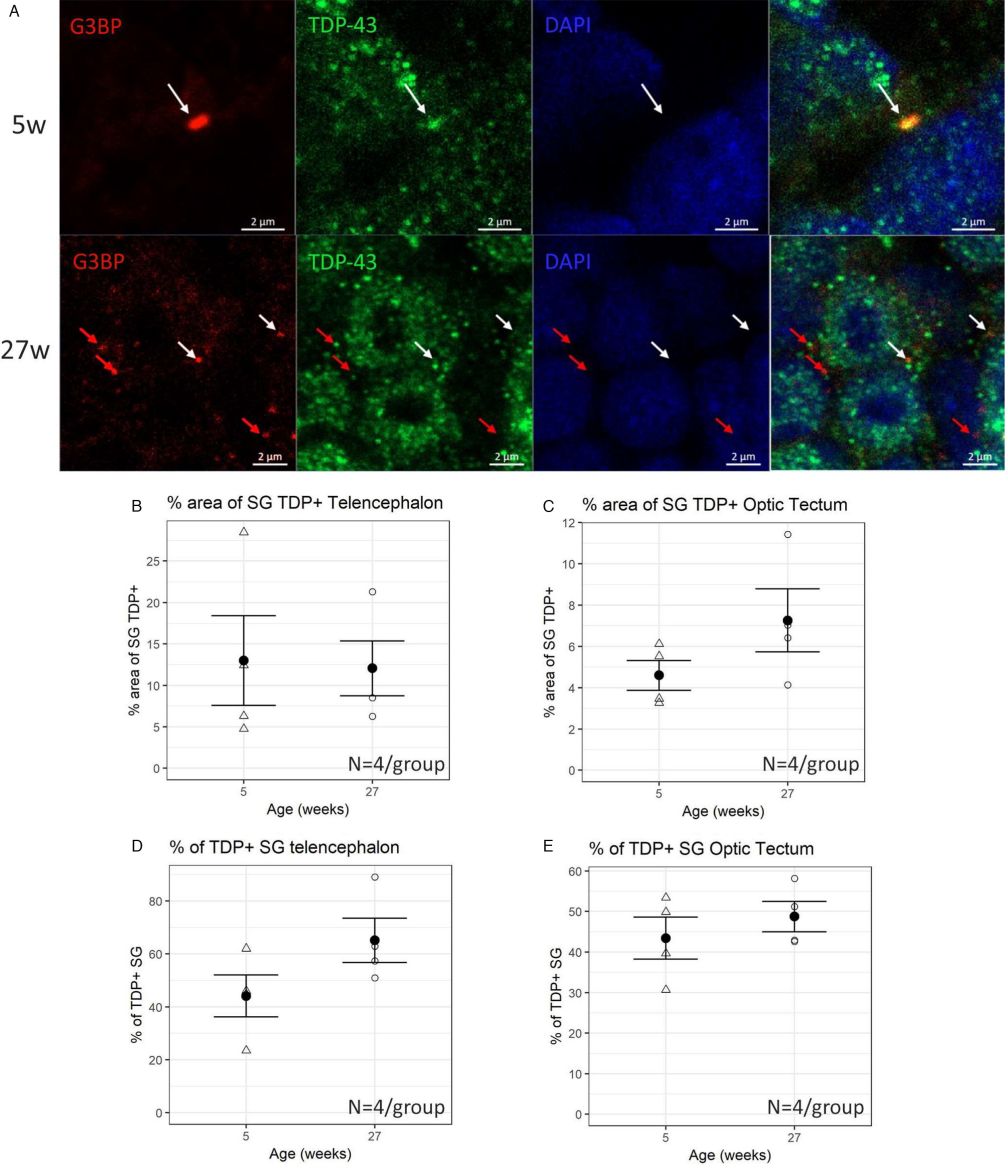
Localization of TDP‐43 in SGs. The presence of SGs both in young (5 weeks) and old (27 weeks) animals was detected. Some SGs colocalize with TDP‐43 (white arrows). Those not colocalizing (red arrows) were located in close proximity of TDP‐43. (b) Quantification of the area of SGs also labeled with TDP‐43 in telencephalon. (c) Quantification of the area of SG also labeled with TDP‐43 in optic tectum. (d) Number of SGs containing TDP‐43 expressed as a percentage in telencephalon. (e) Number of SGs containing TDP‐43 expressed as a percentage in optic tectum

Finally, we investigated the possible colocalization of TDP‐43 with SGs in *N. furzeri* tissues in agreement with our in silico predictions. We performed double immunostaining for *N. furzeri* TDP‐43 and G3BP, a core protein of the SGs often used as a marker (Figure 7a). To check the specificity of an anti‐G3BP antibody, we performed a Western Blot on young (5 weeks) and old (37 weeks) samples (Figure S6) that showed, as expected, a main band just above 50 kDa. We were able to observe the presence of granular structures double‐labeled for G3BP and TDP‐43 in samples of both young and old animals. Some SGs colocalized with TDP‐43 (Figure 7a, white arrows). Those not colocalizing (red arrows) were located in close proximity of TDP‐43. This evidence supports the idea that a TDP‐43 involvement in SG formation and regulation is conserved in *N. furzeri*. To support the hypothesis of an increase of stressing conditions during aging, and thus an increased formation of SGs inside the cell, we quantified TDP‐43^+^ SGs in the two brain regions previously analyzed (Figure 7b,d and c,e). Quantification did not show any significant difference between young and old animals, both in terms of area of SGs colocalizing with TDP‐43 and number of granules positive for TDP‐43 (Figure 7b,c and d,e).

Taken together, these exciting findings demonstrated the possibility to study granule formation and model TDP‐43‐related pathologies in *N. furzeri*.

## 3 DISCUSSION

In this manuscript, we laid the foundations for the use of *N. furzeri* as a new animal model to follow TDP‐43 age‐dependent aggregation. Increasing evidence has shown that *N. furzeri* shares molecular, histopathological, and behavioral aging‐related signatures with mammals, making this fish the most promising vertebrate model for research on aging (Cellerino et al., 2016): *N. furzeri* is easily manageable, relatively inexpensive, and has a very limited lifespan (3 to 7 months depending on the strain). It is also an animal that presents clear signs of aging: old fishes appear emaciated, with their spine curved and almost discolored while showing cognitive and locomotor age‐dependent decay at a behavioral level. This makes easy to follow the aging process and relate it to other phenotypes. The possibility to insert human pathogenic mutations in the *N. furzeri* orthologue gene using CRISPR/Cas9 adds up a further advantage in support to this model (Harel et al., 2015).

A bioinformatic analysis demonstrated a remarkably high homology between various *N. furzeri* and mammalian protein sequences. Accordingly, we found an amino acidic identity between TDP‐43 from *N. furzeri* and Hsa_TDP‐43 above 75%. This is not surprising since, on average, about 30% of human genes are duplicated in teleosts (Braasch et al., 2016). Once duplicated, the two paralogs have clearly taken an independent evolutionary trajectory. The homology of both paralogues with the human protein is higher in the N‐terminus but breaks down in the C‐terminus. This preliminary analysis made us to expect similar properties of nucleocytoplasmic transport, RNA and DNA binding, and phase separation.

We used in vitro studies to compare the individual behavior of different regions of the proteins from human and *N. furzeri* using recombinant fragments spanning the C‐termini and the tandem RRM domains. We observed a strong tendency to aggregate of the C‐termini in agreement with what has been observed for the human protein (Capitini et al., 2020; Vega et al., 2019). This region is so aggregation prone that the recombinant human C‐terminus could be obtained in a soluble form only by cleaving off the glycine‐rich region in agreement with previous studies (Capitini et al., 2020). At variance, constructs of the *N. furzeri* C‐termini from the end of RRM2 to the end of the proteins are soluble in *E. coli* expression, which may constitute an advantage in future studies. Here, we observed a remarkably similar behavior of the proteins, with the Nfu_TDP‐43L C‐terminus behaving closer to the human protein.

When the analysis was carried out on the RRM1‐2 constructs, we predictably noticed only minor differences among their aggregation properties according to the sequence conservation. If proteins from organisms as far apart as human and killifish retain a similar tendency to aggregate and misfold, the property must be inherent to the protein and its function.

We analyzed in silico the RNA‐binding specificities of the full‐length proteins, being prepared to observe almost identical profiles. We found instead only a partial overlap of putative partner sequences, with Nfu_TDP‐43L being closer to Hsa‐TDP‐43. While these results await solid experimental confirmation, possibly by iCLIP studies or other cellular screenings, our data suggest an involvement of regions outside the RRM domains in RNA binding that could explain the presence of more than one TDP‐43 in *N. furzeri* and/or different RNA propensities that are reflected by the minute but appreciable differences in the RRM1‐2 sequences.

This second hypothesis was directly backed up by our studies. We tested the effects on the aggregation of the *N. furzeri* RRM1‐2 domains of the RNA12 aptamer known to bind to Hsa_TDP‐43 with affinity in the low nanomolar range (Lukavsky et al., 2013). We have previously demonstrated that the presence of this aptamer is sufficient to inhibit aggregation (Zacco et al., 2019), suggesting RNA as a cellular chaperone of TDP‐43 (Louka et al., 2020). We observed an RNA‐induced appreciable reduction of aggregation also for the *N. furzeri* proteins. Interestingly, the effect is less marked for Hsa_TDP‐43 and Nfu_TDP‐43L than for Nfu_TDP‐43, suggesting a lower affinity. Given the identity of almost all residues in contact with the aptamer as observed in the solution structure of the complex, we suggest that a lower affinity to the aptamer could be caused by the six amino acid insertion between the two RRM domains of Nfu_TDP‐43. A different length of the linker could change the mutual orientation of the two domains and their dynamics that could in turn affect the interaction with RNA. It is interesting to note that the insertion is immediately after R181, the residue associated with a clinically important mutation (Chen et al., 2019). Together, these observations point at a previously unreported important role of the linker between the two RRM domains in RNA recognition.

In parallel to these in silico and in vitro studies, we carried out an ex vivo investigation in animal tissues to test whether TDP‐43 can form aggregates also in the animal and/or behaves differently in young and old fishes. TDP‐43 was previously detected in the total protein extract, and the data reported in Table EV2 of Kelmer‐Sacramento et al. (2020) show that the concentration of TARBP (Nfu_p_1_050638, ENSNFUG00015014290) decreases significantly between 5 and 12 weeks of age (Log2FC = −0.68, q‐value = 0.0008) but increases significantly (Log2FC = 0.58, q‐value = 0.003) between 12 and 39 weeks of age, suggesting that increased protein concentration may favor aggregation.

Using immunofluorescence experiments, we proved that *N. furzeri* cells may show an abnormal distribution of nuclear TDP‐43 that increases markedly during aging. This could be interpreted as a sign of impaired nucleocytoplasmatic transport, which does not come as a surprise, since dysfunction of nucleocytoplasmatic transport is often associated to neurodegenerative diseases and observed in physiological aging (Hutten & Dormann, 2020). TDP‐43 neurotoxicity in neurodegenerative disorders could thus be linked to a loss of function of nuclear TDP‐43 (Xu, 2012) and, more in general, to dysfunction of nucleocytoplasmatic transport. We were able to observe protein aggregates exclusively in old brain tissues. Some of these aggregates were positive to TDP‐43 staining and strictly associated to doughnut‐like cells, as the proof of the ability of TDP‐43 to aggregate in vivo in this model. However, TDP‐43 aggregates seemed to be not exclusive to a single brain region, as proven by the quantification of TDP‐43+ aggregates in two different areas (telencephalon and optic tectum) of the old tissue. This findings allowed us to conclude that *N. furzeri* TDP‐43 is indeed able to form intracellular pathological aggregates in vivo and strengthened the interpretation of doughnut‐like cells as a pathological condition that can, in the future, be used as a marker to identify aged animals. Intranuclear localization of TDP‐43 in doughnut‐like cells was assessed via double immunofluorescence with nucleoporins, constitutive proteins of the nuclear pores. The peculiar internal perinuclear distribution and the strict interaction of TDP‐43 with nucleoporines could be a consequence of altered nucleocytoplasmatic transport.

In addition, we demonstrated colocalization of TDP‐43 and SGs, both in young and old animals, confirming that the interaction between TDP‐43 and SGs is a common feature also in *N. furzeri*. To do so, we performed quantifications of colocalization of TDP‐43 with G3BP (a primary nucleator of SGs in the cell), but we did not observe any significant change between young and old subjects. TDP‐43 presence in SGs is not permanent but dynamically regulated in response to cellular metabolism as described in the literature (Khalfallah et al., 2018; Aulas et al., 2012, Cohen et al., 2011). In a context of persistent cellular stress, TDP‐43 localizes initially to SGs, then detaches and forms insoluble cytoplasmic inclusions (Parker et al., 2012). Inclusions formation has been observed upon overexpression of mutated forms of TDP‐43 with increased cytosolic SG localization or increased aggregation propensity (Besnard‐Guérin, 2020; Cohen et al., 2011; Guo et al., 2011). Based on these experimental observations, we conclude that the amount of TDP‐43 inside SGs is highly dependent on the state of cellular stress; therefore, the quantification inside a tissue depends on the physiological state of the individual cells. Nonetheless, the presence in SG indicates that TDP‐43 has a role in their formation /regulation as observed in mammals.

It has been shown that SGs impair nucleocytoplasmatic transport and that stress granule suppression prevents neurodegeneration in an ALS/FTD model based on C9ORF72 (Zhang et al., 2018). This observation suggests that, by preventing access of TDP‐43 to the nucleus, stress granule formation may be central to the pathogenic mechanisms of ALS. While this is only the first step toward a more detailed determination of the role of TDP‐43 and its aggregates in aging and neurodegeneration, our findings offer brand new possibilities for the study of ALS and other aging diseases. Besides its application for our basic understanding of the disease mechanisms, it will be for instance interesting to test in *N. furzeri* the effect of RNA aptamers against TDP‐43 aggregation, as those described in our previous work (Zacco et al., 2019), having the possibility to correlate aggregation with phenotype in an animal with a short lifespan.

Although it has been shown that TDP‐43 pathology can be detected in asymptomatic human individuals, it is also known that accumulation of TDP‐43 aggregates is strictly associated with cognitive decline (Wilson et al., 2013). Furthermore, it is possible to induce ALS‐like symptoms (i.e., progressive motor weakness and muscle atrophy with fasciculation and regional cytoplasmic mislocalization of TDP‐43) for instance by injection of AAV expressing TDP‐43 into cynomolgus monkeys (Uchida et al., 2012). Although we are not aware of any spontaneous TDP‐43‐related pathology in other animals, a unique experimental model such as *N. furzeri*, which allows to follow the aggregation mechanisms of TDP‐43, remains a valuable resource for a deeper understanding of these pathologies.

In conclusion, our results demonstrate that *N. furzeri* is a powerful model for TDP‐43‐related diseases. The small but interesting differences in the aggregation and RNA‐binding properties of the individual sequences may be favorably exploited in the future to deepen our understanding of protein aggregation, stress granule formation, RNA specificity, and neurodegenerative processes. Further studies will be needed to observe the TDP‐43‐related aging process in *N. furzeri* in a more statistically significant manner and to identify possible modifiers of stress granule dynamics dysregulation in TDP‐43‐related neurodegenerative diseases.

## 4 EXPERIMENTAL PROCEDURES

### 4.1 Recombinant protein production

Recombinant constructs encompassing the RRM1‐2 and C‐terminal domains of Hsa_TDP‐43 (K102–Q269, Q269–H414, and A315–H414, Uniprot entry: Q13148), Nfu_TDP‐43 (K103–P269 and P269–M403, NCBI Ref: XP_015823124.1), and Nfu_TDP‐43L (K103–Q269 and Q269–M405, NCBI Ref: XP_015814443.1) were cloned in a pET‐SUMO plasmid with an N‐terminal hexa‐His tag, a SUMO tag with a modified polylinker site and a cleavable tobacco etch virus (TEV) protease site. The *N. furzeri* sequences were only predicted and could thus be splicing variants as we could not exclude that there might be more isoforms for each gene. cDNA was purchased from GenScript with codon optimization for *E. coli* expression. The plasmids were prepared by the Gibson's assembly strategy (Gibson et al., 2009). The proteins were purified as previously described (Zacco et al., 2019). In short, the plasmids were transformed in NiCo21(DE3) cells and grown overnight up to a 0.7 optical density recorded at 600 nm at 37°C in Luria‐Bertani medium containing 50 μg/ml kanamycin, before inducing expression overnight by 0.5 mM IPTG at 18°C. The cells were collected after centrifugation and lysed by sonication. The soluble fusion proteins were purified by nickel affinity chromatography (Super Ni‐NTA agarose resin, Generon) and eluted from the column with high‐salt phosphate buffer (10 mM potassium phosphate buffer, 150 mM KCL, 2.5 μM TCEP, at pH 7.2 for RRM1‐2 and pH 6.5 for the C‐terminal constructs) with the addition of 250 mM imidazole. The tag was cleaved off by incubating the construct with TEV (at a 1:10 protein to protease molar ratio). All samples were further purified by nickel affinity chromatography. Nonspecifically bound nucleic acids were removed by performing heparin affinity chromatography (HiTrap Heparin HP; GE Healthcare), on an Äkta system. This enables simultaneous observation of the absorbance at 260 and 280 nm. The proteins were eluted using a high‐salt gradient (1.5 M KCl in 10 mM potassium phosphate buffer). The core proteins were obtained after size‐exclusion chromatography with a HiLoad 16/60 Superdex75 prep grade in 10 mM potassium phosphate buffer, 15 mM KCl, 2.5 μM TCEP pH 7.2 for RRM1‐2 and pH 6.5 for the C‐terminal constructs, aliquoted, flash‐frozen, and stored at −20°C. The purity of the proteins was checked by SDS‐PAGE gels.

### 4.2 ThT and proteostat aggregation assays

Aggregation kinetics of the isolated proteins were determined by the Thioflavin T (ThT)‐binding assay followed by a BMG FLUOstar Omega microplate reader in Greiner Bio‐One CELLSTAR plates. When not carried out immediately after purification, protein aliquots were rapidly thawed from the freezer, spun down, and filtered with a 0.2 µm syringe filter before each assay to ensure removal of any contingent preformed oligomer. The resulting concentration was determined. The solutions were diluted to final concentrations of 10 µM for the RRM1‐2 domains and 6 µM for the C‐termini. The ThT concentration was 20 µM. The plate was sealed with an optic seal to avoid evaporation. The experiments were carried out at 37°C by recording the fluorescence intensity of ThT as a function of time for 48 h using 430 nm as the excitation wavelength and 485 nm for the emission. Experiments in which the fusion protein was cleaved directly in the plate were carried out by adding TEV protease directly to the protein samples using 1:10 enzyme to protein molar ratios. Experiments carried out in the presence of RNA used the fluorescent dye ProteoStat. The aptamer was added to the protein in an RNA to protein 2:1 molar ratio using 10 µM concentrations of RRM1‐2. The excitation wavelength was set at 500 nm and the emission at 600 nm.

### 4.3 CD measurements

CD spectra were recorded on 10 µM samples in phosphate buffer and 15 mM KCl with a JASCO‐1100 spectropolarimeter. The spectra were acquired in 1 mm path length quartz cuvettes under a constant N_2_ flush at 4.0 L/min. All datasets were an average of thirty scans. Far‐UV (190–260 nm) spectra were recorded at 20°C and then gradually heated to 90°C (1°C/min) for the determination of the melting temperature (Tm). The data were plot and the Tm calculated according to literature (Greenfield, 2006). Control CD spectra were acquired after the temperature was brought back to the original 20°C. The spectra were corrected for the buffer signal and expressed as mean residue molar ellipticity θ (deg × cm^2^ × dmol^−1^).

### 4.4 Animal housing and tissue preparation

The protocols of fish maintenance were carried out in accordance with all animal use practices approved by the Italian Ministry of Health (Number 96/2003a) and the local animal welfare committee of the University of Pisa. All experimental procedures were managed, following the prescription of the European (Directive 2010/63/UE) and Italian law (DL 26/04‐03‐2014).

*Nothobranchius furzeri* fishes were hatched and housed locally in a Tecniplast Zebtech system with automatized water flow, pH, and salinity control. All animals were hatched, fed, and maintained as previously described (Terzibasi Tozzini et al., 2008). Eggs were maintained on wet peat moss at room temperature in sealed Petri dishes. When embryos had developed, eggs were hatched by flushing the peat with tap water at 16–18°C. Embryos were scooped with a cut plastic pipette and transferred to system tank. Fry larvae were fed with newly hatched Artemia nauplii for the first 2 weeks and then weaned with finely chopped Chironomus larvae. The system water temperature was set at a constant 27°C. At the defined age, fish were sacrificed via anesthetic overdose (Tricaine, MS‐222), in accordance with the prescription of the European (Directive 2010/63/UE) and Italian (DL 26/04‐03‐2014) laws. Brains were immediately extracted under a stereomicroscope and fixed overnight in a solution of PFA 4% in PBS.

Brains from 5‐ and 27‐week‐old animals were microdissected and processed for immunohistochemistry protocols: tissues were first fixed with paradormaldehyde (PFA) 4% in PBS (O/N at 4°C) and cryoprotected with sequential immersions in sucrose 20% and 30% until the tissue precipitated to the tube bottom (a minimum of 6 h/each steps). The tissues were singularly included in Tissue‐Tek^®^O.C.T.™ Compound (Sakura), and 25 tick sections were cut on Superfrost Plus adhesion slides (Thermo Fisher Scientific).

### 4.5 3D visualization of doughnut‐like cells in *N. furzeri*

We used the AbSca/e method to visualize the cells presenting an abnormal nuclear distribution of TDP‐43 in *N. furzeri*. This is a clearing and staining approach for whole‐mount tissues that is faster than the original Scale methods and avoids tissue expansion and preserves lipids (Hama et al., 2015). The technique was adapted to the considerably smaller size of *N. furzeri* brains (Tables S1 and S2). In short, the brains were fixed after dissection in 4% PFA overnight and adapted with a S0 solution for 18 h at 37°C. The samples were then permeabilized with sequential incubation in A2‐B4(0)‐A2 solutions at 37°C. After the permeabilization steps, samples were de‐Scaled through incubation in PBS for 6 h at room temperature, followed by incubation with polyclonal TDP‐43 primary antibody in an AbSca/e solution for 3 days at 4°C, rinsed twice in AbSca/e for 2 h, and incubated with a secondary antibody for 18 h at 4°C. The samples were rinsed for 6 h in AbSca/e and subsequently in the AbRinse solution twice for 2 h. After a refixation step in 4% PFA for 1 h and a rinse in PBS for an additional hour, samples were finally clarified in Sca/e S4 for 18 h at 37° and maintained in Sca/eS40 at 4° until imaging. The Sca/eS4 solution was used as imaging medium.

### 4.6 Immunofluorescence and proteostat aggresome assay

We performed immunofluorescence experiments on cryosections of 25 microns and proceeded as previously described (Terzibasi Tozzini et al., 2012). Briefly, we washed the sections in PBS to remove the cryoembedding medium. We then performed an acid antigen retrieval step (10 mM trisodium citrate dehydrate, 0.05% tween, at pH 6) and (when required) stained the section with the ProteoStat Aggresome Detection Kit (Enzo Life Sciences Inc.) as previously described (Shen et al., 2011). We applied a solution 1:2000 of Aggresome dye in PBS for 3 min, rinsed the samples with PBS, and left the sections immersed in 1% acetic acid for 40 min. We applied the blocking solution (5% BSA, 0.3% Triton‐X in PBS) for 2 h. Primary antibodies at proper dilution were added in 1% BSA, 0.1% triton in PBS, and left overnight at 4°C (Table S3). The day after, we applied secondary antibodies (1:400 dilution) in the same solution. After 2 h at room temperature, slides were washed three times with PBS and mounted with Fluoroshield DAPI mounting medium (Sigma‐Aldrich).

For all further quantifications, slides from sets of 4–5 animals per group (young and old) were used.

### 4.7 Western blots

We performed Western Blots both on the purified TDP‐43 domains and on brain extracts to assess recognition of the polyclonal anti‐TDP‐43 antibody and check specificity. We used 35 µg of purified protein samples and ran them in a 12% SDS‐PAGE gel for 2 h at 100V. Afterward, the samples were blotted on a nitrocellulose membrane for 1 h at 100V. The membrane was incubated with the primary antibody overnight at 4°C and then incubated with the secondary antibody for 1 h at room temperature. The membranes were imaged with a Chemidoc XRS scanner using the Quantity one Biorad software.

For the Western Blots in *N. furzeri* brains, these were extracted, immediately stored at −80° and then homogenized for 30 s using the GelD2 buffer with the addition of protease and phosphatase inhibitors. After centrifugation for 10 min at 28000g, the supernatant was collected, quantified using the BCA kit (Thermofisher), and stored at −80° for further use. To perform the Western Blot, we used 20 µg of protein extract derived from two samples per age and ran them in a 10% SDS‐page gel for 45 min at 150 V. Blotting and incubation procedures were the same as the ones described above.

### 4.8 Quantification of aggresome and G3BP colocalization with TDP‐43

To quantify the colocalization of TDP‐43 with G3BP and aggresome staining, we acquired high‐resolution images (2048 × 2048 px) at a Zeiss confocal microscope using a 63× objective. For each animal, we analyzed three images per area (telencephalon, optic tectum). We used the Spot detector algorithm in the open license software Icy (http://icy.bioimageanalysis.org/) for spot analysis. In both cases, we imposed a threshold to isolate the SGs or aggregates and launched the algorithm to identify the granules of TDP‐43 comprised inside the SG/aggregates. We exported the data regarding the number of granules detected (both SG/aggregates and TDP‐43) and the area of the granules. We divided the number of TDP‐43 granules for the number of SG/aggregates to obtain the percentage of SG/aggregates containing TDP‐43 and the area of TDP‐43 for the area of SG/aggregates.

### 4.9 Computational tools

*cat*GRANULE software (http://service.tartaglialab.com/new_submission/catGRANULE) was used to predict the protein tendency to phase separate (Bolognesi et al., 2016). *cat*RAPID *signature* was used to predict the propensity of TDP‐43 to interact with RNA and identify RNA‐binding domains (Livi et al., 2016). *cat*RAPID *omics* was employed to compute the binding targets in the human transcriptome (Agostini et al., 2013).

## CONFLICT OF INTEREST

The authors declare no conflict of interest.

## AUTHOR CONTRIBUTIONS

AL, JR, and BE took care of the experimental in vitro experiments, SB carried out all the work on animal samples, GGT is responsible for the bioinformatic analysis, AC is the promoted of the Furzeri project, and ETT and AP supervised the work. All authors contributed to early versions of the manuscript, AP finalized the manuscript, which was then scrutinized by all co‐authors.

## Supporting information

Supplementary Material

Video S1

Video S2

## Data Availability

The experimental data may be available upon request to the authors. The *cat*Granule results are available at https://tinyurl.com/y45hp82b. The *cat*RAPID *signature* results can be retrieved from https://tinyurl.com/y3ondta2, https://tinyurl.com/y3w57dsg, and https://tinyurl.com/y5a6rb3a for Hsa_TDP4, Nfu_TDP‐43L, and Nfu_TDP‐43, respectively. The catRAPID *omics* results are available at https://tinyurl.com/y2swjuxl for Hsa_TDP‐43, Nfu_TDP‐43L, Nfu_TDP‐43, and *N. furzeri* methyltransferase (A0A1A8VE13 or TFB2M). The GU‐rich sequences retrieved can be found at https://tinyurl.com/y5jd8wm5.
