# Supplementary material for: New lessons on TDP‐43 from old N. furzeri killifish
Source: Aging Cell. 2021 Dec 23;21(1):e13517. doi: 10.1111/acel.13517 (PMC8761016; doi:10.1111/acel.13517)
Supplement: Supplementary file 1 — Supplementary Material [file ACEL-21-e13517-s001.docx]

**Supplementary Materials**

**New lessons on TDP-43 from old *N. furzeri* killifish**

Alexandra Louka^1+^, Sara Bagnoli^2+^, Jakob Rupert^1,3^, Benjamin Esapa^1^, Gian Gaetano Tartaglia^3,4^, Alessandro Cellerino^2,6^, Annalisa Pastore*^1^, Eva Terzibasi Tozzini*^2,5^

^1^UK-DRI Centre at the Maurice Wohl Institute, Department of Clinical and Basic Neuroscience, King’s College London, 125 Coldharbour Lane, SE5 9NU, London, UK

^2^Bio@SNS,^,^ Scuola Normale Superiore, Piazza dei Cavalieri, Pisa, Italy

^3^Universita’ di Roma “La Sapienza”, Rome, Italy

^4^Istituto Italiano di Tecnologia, Rome, Italy

^5^Stazione Zoologica Anton Dohrn (SZN), Naples, Italy

^6^Leibniz Institute on Aging, Fritz Lipmann Institute, Jena, Germany


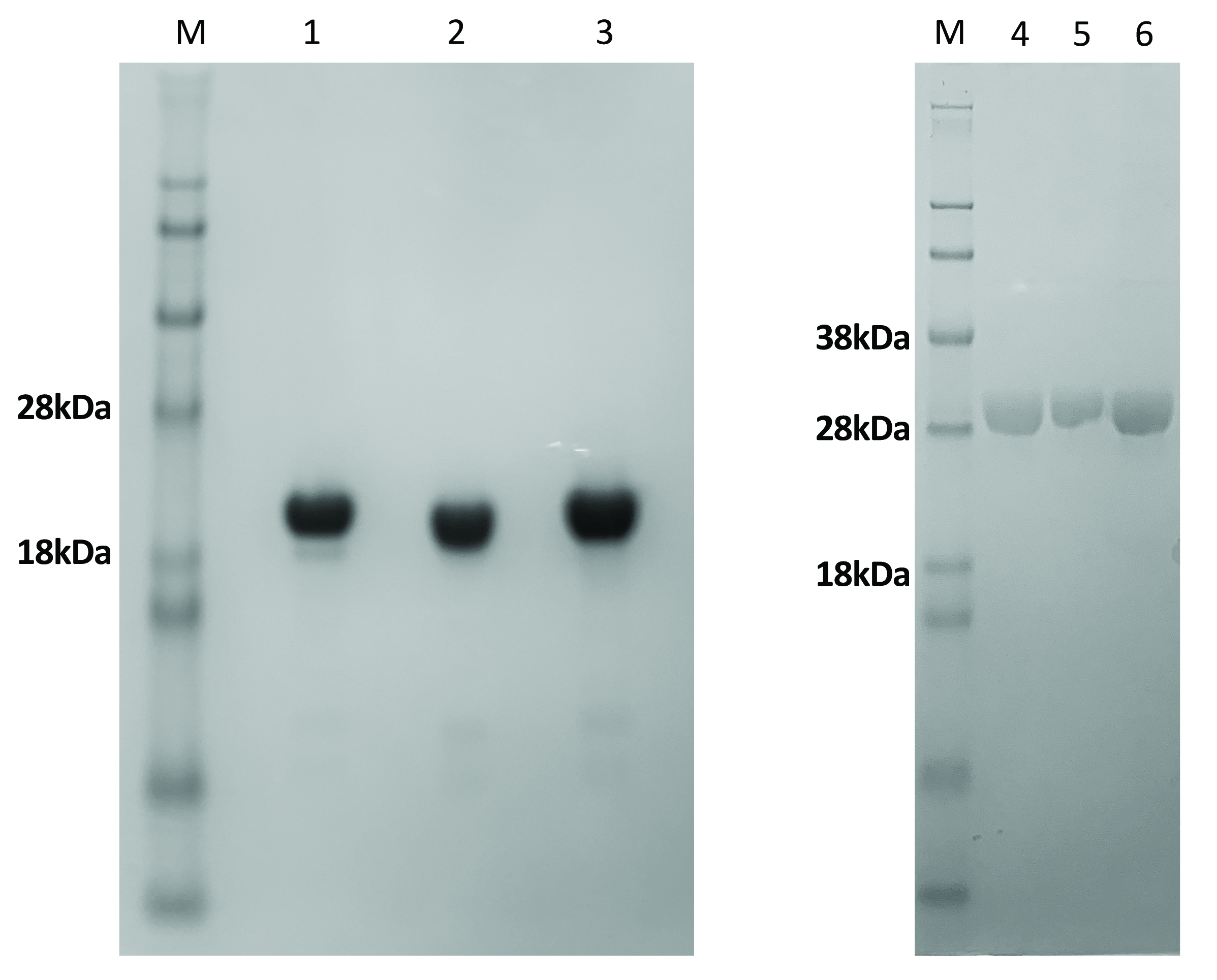


**Figure S1** – Coomassie-stained 12% SDS–PAGE indicating protein purity. The expected molecular weights are 19 kDa for the RRM1-2 domain constructs and 28 kDa for the SUMO C-terminal domain constructs. Lane M: Blue pre-stained protein standards. Lane 1: RRM1-2 from Hsa_TDP-43. Lane 2: RRM1-2 from Nfu_TDP-43. Lane 3: RRM1-2 from Nfu_TDP-43L. Lane 4: SUMO C-terminus from Hsa_TDP-43. Lane 5: SUMO C-terminus from Nfu_TDP-43. Lane 6: SUMO C-terminus from Nfu_TDP-43L.


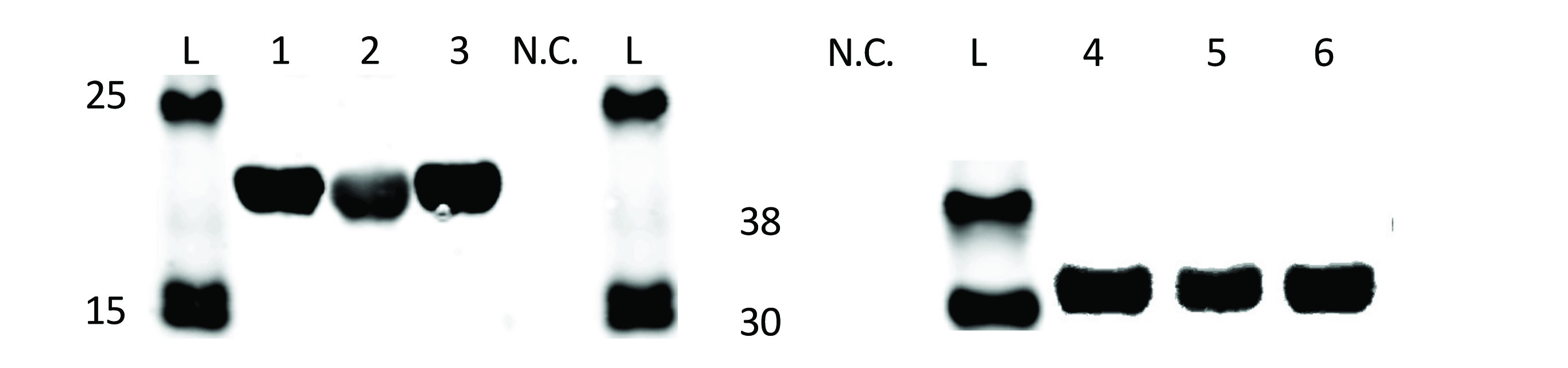


**Figure S2** – Western blot using the (Proteintech) rabbit polyclonal antibody. Lane L: Pre-stained protein ladder. Lane1: RRM1-2 from Hsa_TDP-43. Lane2: RRM1-2 from Nfu_TDP-43. Lane 3: RRM1-2 from Nfu_TDP-43L. Lane4: SUMO C-terminus from Hsa_TDP-43. Lane 5: SUMO C-terminus from Nfu_TDP-43. Lane 6: SUMO C-terminus from Nfu_TDP-43L. Lane NC: Negative Control.


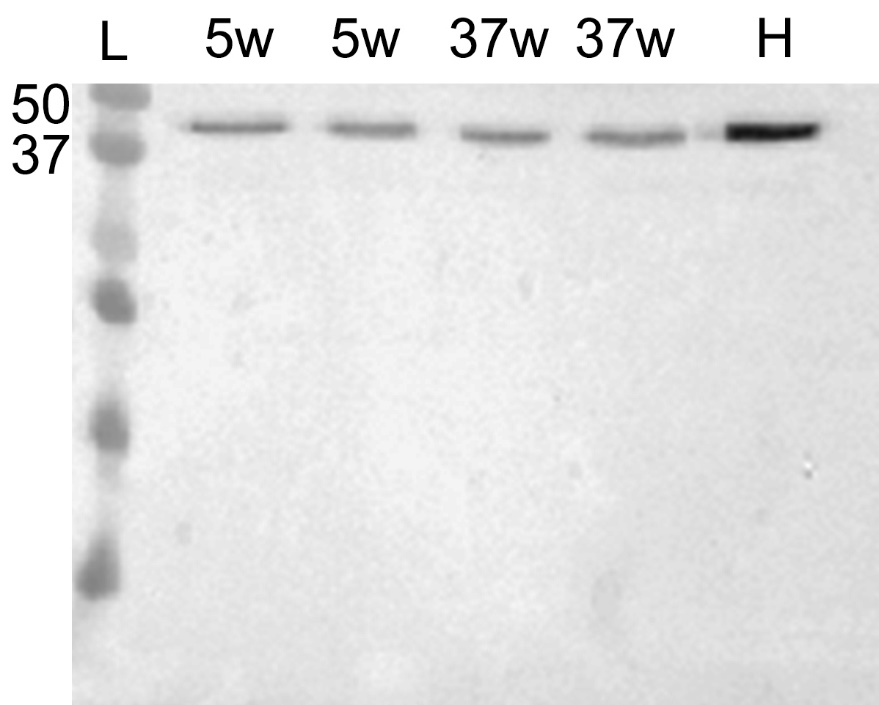


**Figure S3 –**TDP-43 Western Blot of *N. furzeri* whole brain extracts. 12% SDS page for TDP-43. We used Hela cells extract as positive control (line H). *N. furzeri* sample lanes are loaded with 60 µg of whole brain protein extract, HeLa sample line is loaded with 40 µg of lysate. L: ladder. 5w: samples from 5 weeks-old animals. 37w: samples from 37 weeks-old animals, H: samples from HeLa cells lysate.

**Figure S4** – Presence of doughnut-like cells in the optic tectum of old (27 weeks) animals. Z1, 2, 3 and 4 represent four consecutive z planes each acquired at 1 μm step from the previous. White arrowheads indicate doughnut-like cells, red arrowheads indicate normal distribution cells. The scale bar indicates 10 μm.

**
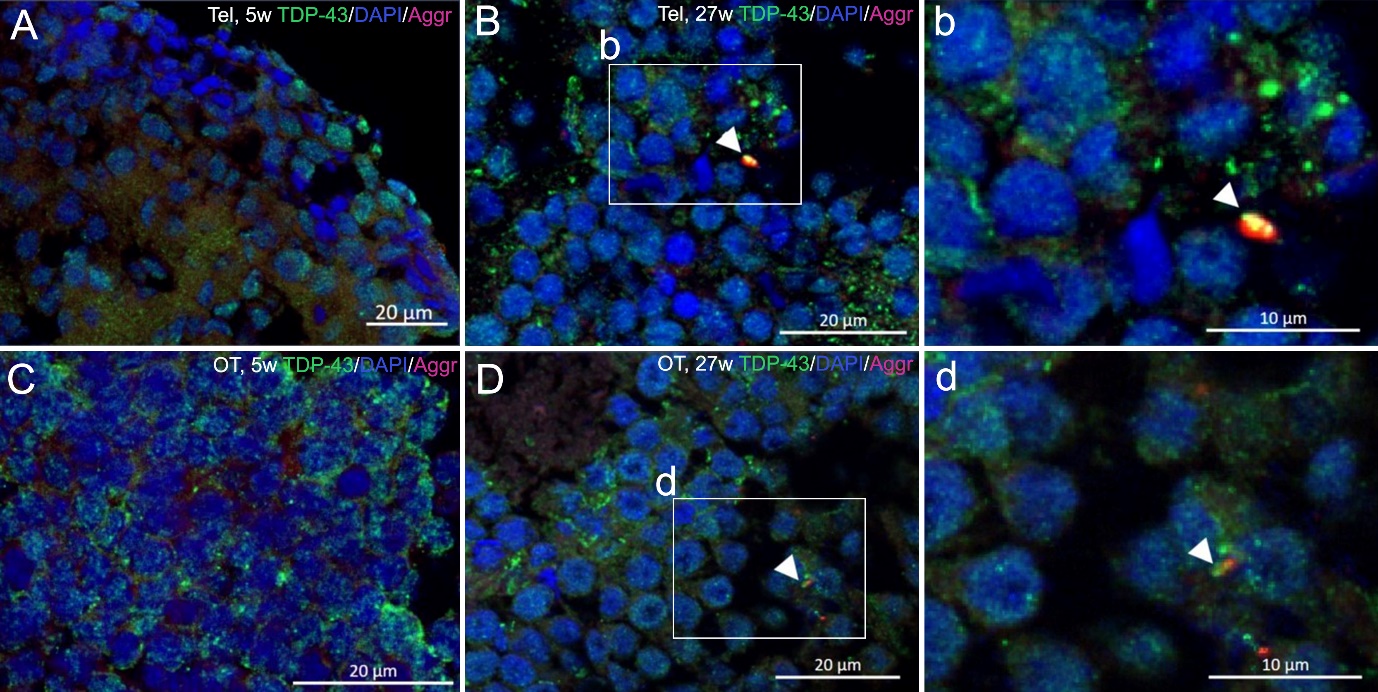
**

**Figure S5** – Immunostaining of TDP-43 and co-localization with protein aggregates in old versus young *N. furzeri* brains. Protein aggregates were stained using aggresome dye (red), while TDP-43 was labelled in green. No trace of aggregation was detectable in young samples (panels A,C). We observed aggregates only in old brains, both in telencephalon (panels B,b, white harrowheads) and optic tectum (panels D,d, white harrowheads).The aggregates were localized mainly near doughnut-like cells (b/d magnifications, white arrowheads).


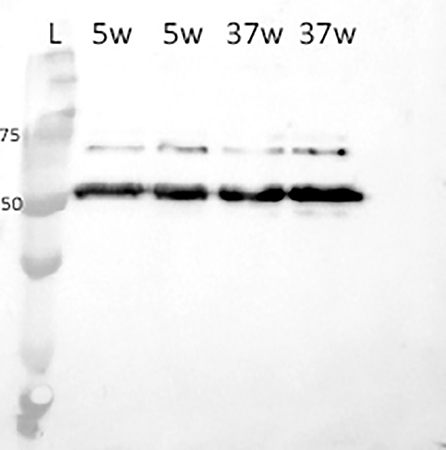


**Figure S6 –** Anti-G3BP Western Blot of *N. furzeri* whole brain extracts on a 10% SDS page gel. Each sample lane is loaded with 20 µg of whole brain protein extract. L: ladder. 5w: samples from 5 weeks-old animals. 37w: samples from 37 weeks-old animals.

**Table S1.** AbSca/e staining procedure. This is the procedure we adapted from Hama et al., 2015 for clearing and staining of *Nothobranchius furzeri* brains. In the table are reported the solution required for each step with the time and temperature of incubation. ON: over night, RT: room temperature.

| **Step** | **Solution** | **Timing (approx.)** | **Temperature (°C)** |
| --- | --- | --- | --- |
| Fixation | 4% PFA | ON | 4 |
| Adaptation | Sca/e S0 | 18h | 37 |
| Permeabilization | Sca/e A2 | 36h | 37 |
|  | Sca/e B4(0) | 24h | 37 |
|  | Sca/e A2 | 12h | 37 |
| Descaling | PBS | 6h | RT |
| Immunostaining | AbSca/e + primary antibody | 3 days | 4 |
|  | AbSca/e | 2h (2x) | RT |
|  | AbSca/e + secondary antibody | 18h | 4 |
| Wash | AbSca/e | 6h | RT |
| Rinse | AbRinse | 2h (2x) | RT |
| Refixation | 4% PFA | 1h | RT |
| Wash | PBS | 1-2h | RT |
| Clearing | Sca/e S4 | 18h | 37 |
| Mounting | Sca/e S4 |  | 4 |

**Table S2.** Composition of the solution used for the AbSca/e protocol. The composition is taken as indicated in Hama et al., 2015.

| **Ingredients** | **Sca/eA2** | **Sca/e B4(0)** | **Sca/eS0** | **Sca/eS4** | **AbSca/e** | **AbRinse** |
| --- | --- | --- | --- | --- | --- | --- |
| D-(-)-sorbitol (w/v%) | - | - | 20 | 40 | - | - |
| Glycerol (w/v)% | 10 | - | 5 | 10 | - | - |
| Urea (M) | 4 | 8 | - | 4 | 0.33 | - |
| Triton-X-100 (w/v)% | 0.1 | - | - | 0.2 | 0.5 | 0.05 |
| Methyl-β-cyclodextrin (mM) | - | - | 1 | - | - | - |
| γ-Cyclodextrin (mM) | - | - | 1 | - | - | - |
| N-acetyl-L-hydroxyproline (w/v)% | - | - | 1 | - | - | - |
| DMSO (w/v)% | - | - | 3 | 25 | - | - |
| PBS | - | - | 1x | - | - | 0.1x |
| BSA (w/v)% | - | - | - | - | - | 2.5 |

**Table S3.** List of antibodies utilized in the immunofluorescence experiments.

| **Antibody** | **Product type** | **Working dilution** | **Company (Cat N.)** |
| --- | --- | --- | --- |
| Anti-TDP-43 | Rabbit Polyclonal | 1:1200 | Proteintech (10782-2-AP) |
| Anti-G3BP | Mouse Monoclonal | 1:500 | AbCam (ab56574) |
| Anti-Nup | Mouse Monoclonal | 1:100 | AbCam (ab24609) |
